# Effects of Levetiracetam on the Serum C-Reactive Protein in Children With Epilepsy: A Meta-Analysis

**DOI:** 10.3389/fphar.2022.810617

**Published:** 2022-04-20

**Authors:** You-Feng Zhou, Yan Huang, Guang-Hua Liu

**Affiliations:** ^1^ Department of Pediatrics, Fujian Provincial Maternity and Children’s Hospital, The Affiliated Hospital of Fujian Medical University, Fuzhou, China; ^2^ Department of Children’s Healthcare, Fujian Provincial Maternity and Children’s Hospital, The Affiliated Hospital of Fujian Medical University, Fuzhou, China

**Keywords:** children, epilepsy, crp, levetiracetam, meta-analysis

## Abstract

This meta-analysis aims to evaluate the effect of levetiracetam on serum C-reactive protein (CRP) in children with epilepsy. Articles published up to April 15, 2021 were searched from Google Scholar databases, PubMed, Science Direct, Springer, Wiely, NIH and Baidu Scholar databases to analyzed the difference of serum CRP in epilepsy children compared to healthy controls, and the effect of levetiracetam on serum CRP in children with epilepsy was also assessed. All the included studies met the inclusion criteria. 103 publications were selected and eight articles were included in this study with sample size *n* = 246. The serum CRP level in childhood epilepsy was significantly higher than the healthy controls (pooled standardized mean difference (SMD): 6.930, 95% CI: 2.716–11.143, z = 3.22, *p* < 0.01). A significant level of between-study heterogeneity was found (τ^2^ = 17.911, Chi^2^ = 148.67, df = 3, *p* < 0.01, I^2^ = 98.0%). Besides, serum CRP level was significantly decreased by the treatment of levetiracetam in childhood epilepsy (pooled SMD: 3.505, 95% CI: 1.638–5.373, z = 3.68, *p* < 0.01). A significant level of between-study heterogeneity was found (τ^2^ = 4.346, Chi^2^ = 97.17, df = 4, *p* < 0.01, I^2^ = 95.9%). The funnel plot showed there was no significant publication bias in the meta-analysis. Serum CRP levels are upregulated in childhood epilepsy and reduced by levetiracetam in children with epilepsy.

## Introduction

Epilepsy is one of the common neurological disorders, caused by abnormal discharge or synchronization of brain neurons. Epilepsy usually complicates with a series of neurological, psychological, cognitive and sociological complications (Beghi, 2020). Up to 50% children with epilepsy suffer the onset of epilepsy in childhood with a serious impact on their life and development (Zack and Kobau, 2017). Therefore, it is of great significance to discover potential therapeutic drugs against childhood epilepsy.

In the last decades, antiepileptic drugs (AEDs) have been widely used for the treatment of epilepsy in clinic practice (Liguori et al., 2018). As the second-generation of AEDs, levetiracetam shows less adverse effects and good pharmacodynamics in the treatment of epilepsy compared with the other kinds of AEDs (Grinspan et al., 2018). As reported, levetiracetam is has been considered as the first-line antiepileptic medication for preterm infants because of the satisfactory efficacy and safety (Mert and Teki̇nOrgun, 2020). The effect of levetiracetam is also evaluated in a retrospective study on childhood epilepsy, and the findings suggest that intravenous administration of levetiracetam may be effective in various clinical situations (Goraya et al., 2008). Accumulating evidence reveals that there is a positive correlation between epileptogenesis and brain inflammation. Epilepsy increases the expression of essentially inflammatory factors and aggregates brain damage, which in turn induces the recurrence of epilepsy (Kim et al., 2010). Besides, an animal study reveals the antihyperalgesia effect indueced by levetiracetam in rats, indicating the potentials to relieve inflammatoion-induced pain in patients (Micov et al., 2010). Levetiracetam inhibits the blood-brain barrier (BBB) failure associated with angiogenesis and pro-inflammatory responses, that is to say, levetiracetam might exhibit neuroprotection against BBB dysfunction through restraining angiogenesis and inflammatory response in epilepsy (Itoh et al., 2016). Several studies suggest elevated C-reactive protein (CRP) in patients with epilepsy. Obvious increases of serum CRP and TNF-α are found in patients with epilepsy and reduced levels of CRP and TNF-α are shown after the treatment of carbamazepine combined with vitamin B12 (Zhou et al., 2018). A meta-analysis revels a significant increase of serum level of CRP in epilepsy patients, suggesting an obvious association between inflammation and epilepsy (Zhong et al., 2019). Compared with routine medication, the combined therapy of sodium valproate and levetiracetam exhibits better efficacy with less adverse reactions in children with epilepsy, in addition, the expressions of inflammatory indicators, including IL-6, hs-CRP and IL-2, are suppressed after the combined treatment (Liu et al., 2020). Due to the limited number of studies on childhood epilepsy receiving levetiracetam therapy and the small sample size, the effect of levetiracetam on CRP in children with epilepsy is uncertain. Hence, we conducted an overview of all studies with high quality on the effect of levetiracetam on serum CRP in children with epilepsy. The credibility of these studies were assessed and efficacy of levetiracetam on serum CRP was confirmed.

In this study, we performed a meta-analysis to investigate the effect of levetiracetam on CRP level in children with epilepsy. It was observed that levetiracetam could significantly decrease the serum level of CRP in childhood epilepsy. This study might give deeper insights to the regulation of levetiracetam for serum CRP level in childhood epilepsy.

## Materials and Methods

### Publication Search Strategy

All the related published researches were obtained from Google Scholar databases, PubMed, Science Direct, Springer, Wiely, NIH and Baidu Scholar databases, and the last search was conducted on April 15, 2021. The following search terms were used (Pediatric, children, infant, childhood, juvenile or adolescent) (seizure or epilepsy) (C-reactive protein or CRP) and levetiracetam. There were no restrictions for published time. Reference lists of related publications and review articles were also studied to look for potential relevant researches. This study followed Preferred Reporting Items for Systematic Review and Meta-Analysis (PRISMA) guidelines. This study didn’t register with PROSPERO and this will be completed in future work.

### Inclusion and Exclusion Criteria

Two independent observers reviewed studies and extracted data for qualification examination according to the predefined criteria. The inclusion criteria for the studies were as follows: 1) observational studies; 2) children were diagnosed with epilepsy aged 0–18 years; 3) childhood epilepsy were treated with levetiracetam with/without comparison to healthy controls; 4) serum level of CRP in childhood epilepsy or healthy controls were analyzed; 5) The full-text of the study could be obtained; 6) studies written in English or Chinese. Exclusion criteria were as follows: 1) case report; 2) animal experiments; 3) duplicated articles or data.

### Study Selection and Data Extraction

The extracted data included authors, publication year, country, trial design, sample size, patient demographics, dosage and age, ratio of male/female, length of treatment, type/syndrome of epilepsy, controlled group interventions and serum level of CRP were collected.

### Statistical Analysis

All statistical analyses were performed using STATA 15.0 (STATA, College Station, TX, United States ). RRs and 95% CIs were calculated with fixed-effect or random-effect models (depended on heterogeneity). Fixed-effects model was selected if heterogeneity among studies didn’t exist; otherwise, a random-effects model was used. The Q statistic *p* < 0.10 or I^2^ >50% was considered to be significant heterogeneity. The changes of serum CRP were analyzed in childhood epilepsy before and after the treatment. Besides, the difference of serum CRP between childhood epilepsy and healthy controls was also analyzed. The publication bias was evaluated by funnel plot and Egger’s test. *p* < 0.05 was considered to be statistically significant.

## Results

### Search Results and Study Characteristics

**FIGURE 1:**
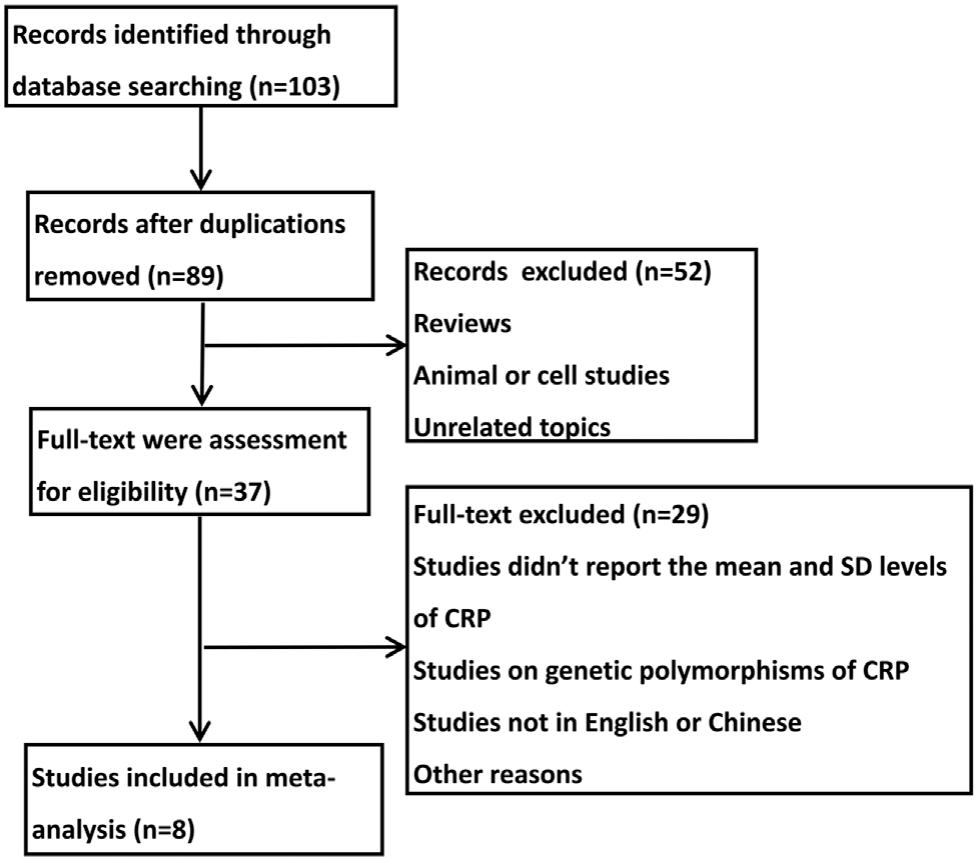
Flowchart of studies selected for review and inclusion for meta-analysis.

As shown in Figure 1, in the initial stage, 103 potential relevant publications were identified, of which 14 publications were excluded after duplication check. After reviewing the abstracts, 52 references were excluded. In the remaining 37 full articles, 29 full texts were excluded for lacking mean and SD levels of CRP, studying genetic polymorphisms of CRP or not presenting in English or Chinese. Thus, data were finally extracted from eight studies (Li, 2012; Jiang, 2013; Huang et al., 2014; Ishikawa et al., 2015; Wang et al., 2018; Nishiyama et al., 2019; Zheng et al., 2019; Chen, 2020) (*n* = 246). All selected studies analyzed the serum level of CRP in children with epilepsy or the healthy controls.

As shown in Table 1, the included studies were published from 2012 to 2020 with a number of cases ranged from 12 to 52. Among these studies, six studies were conducted in China and the other two studies were performed in Japan. All the studies detected serum level of CRP before or after the treatment of levetiracetam in childhood epilepsy or compared the serum level of CRP between childhood epilepsy and healthy controls.

**TABLE 1:** Characteristics of studies included in the meta-analysis.

Study	Published year	Country	Study Type	Case	Age years	Sex male:femal	Type/Syndrome of Epilepsy	Outcome	Method
Li (Li, 2012)	2012	China	Prosp	30	7.9 ± 3.7	18/12	Primary/secondary	Serum hs-CRP level	Immune turbidimetry
Jiang (Jiang, 2013)	2013	China	Prosp	30	7.9 ± 2.3	19/11	Primary	Serum hs-CRP level	ELISA
Huang (Huang et al., 2014)	2014	China	Prosp	36	5.4 ± 2.2	17/19	Primary/secondary	Serum CRP level	Immune turbidimetry
Ishikawa (Ishikawa et al., 2015)	2015	Japan	Prosp	12	4.5 ± 2.6	7/5	Primary/secondary	Serum hs-CRP level	Nephelometry
Wang (Wang et al., 2018)	2018	China	Prosp	39	7–14	22/17	N/A	Serum hs-CRP level	ELISA
Zheng (Zheng et al., 2019)	2019	China	Prosp	35	5.4 ± 2.9	14/21	N/A	Serum CRP level	Immune turbidimetry
Nishiyama (Nishiyama et al., 2019)	2019	Japan	Prosp	12	9.2 ± 2.8	6/6	BECT/Occipital epilepsy/Frontal lobe epilepsy/other focal epilepsy	Serum CRP level	ELISA
Chen (Chen, 2020)	2020	China	Prosp	52	6.4 ± 2.7	30/22	N/A	Serum hs-CRP level	ELISA

BECT: benign epilepsy of childhood with centrotemporal spikes.

### Comparison of Serum Level of CRP Between Children With Epilepsy and the Healthy Controls

**FIGURE 2:**
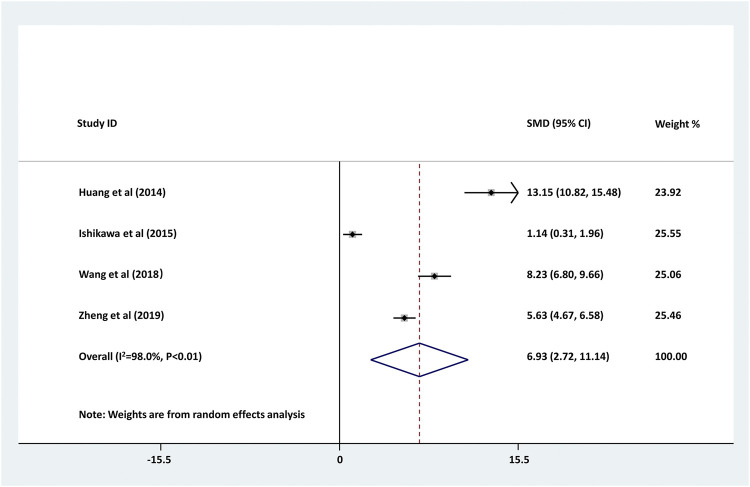
Forest plot of studies comparing serum CRP levels between childhood epilepsy and the healthy controls. Increased serum CRP was found in childhood epilepsy (pooled SMD: 6.930, 95% CI: 2.716–11.143, z = 3.22, *p* < 0.01). The between-study heterogeneity was large (τ^2^ = 17.911, Chi^2^ = 148.67, df = 3, *p* < 0.01, I^2^ = 98.0%).

As shown in Figure 2, four studies compared the serum level of CRP between childhood epilepsyand the healthy controls. The result showed that serum CRP level in childhood epilepsy was significantly higher than that in healthy controls (pooled SMD: 6.930, 95% CI: 2.716–11.143, z = 3.22, *p* < 0.01). Additionally, the between-study heterogeneity was large (τ^2^ = 17.911, Chi^2^ = 148.67, df = 3, *p* < 0.01, I^2^ = 98.0%).

### The Changes of Serum CRP Levels in Childhood Epilepsy Treated With Levetiracetam

**FIGURE 3:**
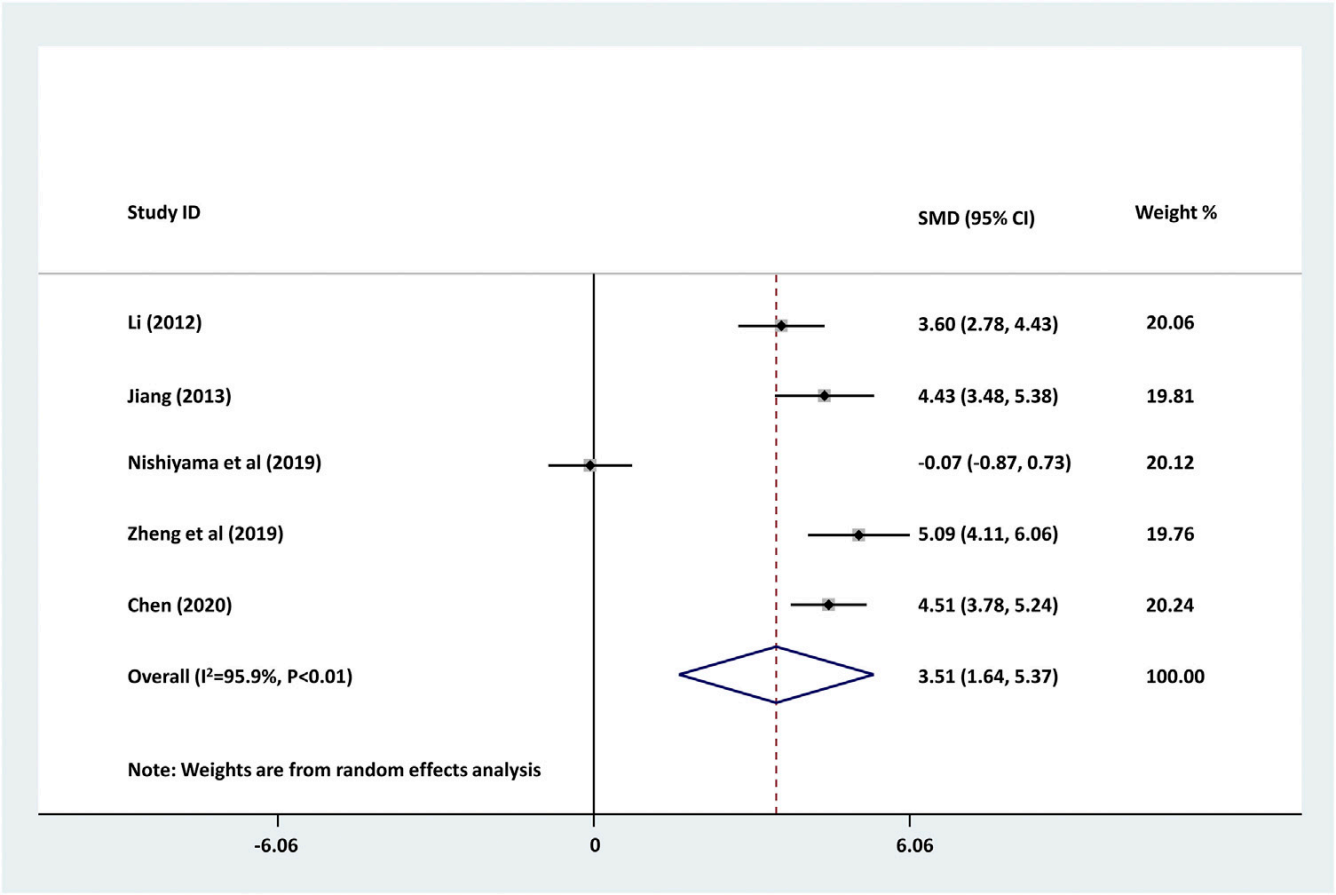
Forest plot of studies detecting the serum changes of CRP levels before and after the treatment of levetiracetam in childhood epilepsy. Decreased serum CRP was found in childhood epilepsy treated with levetiracetam. The between-study heterogeneity was large (τ^2^ = 4.346, Chi^2^ = 97.17, df = 4, *p* < 0.01, I^2^ = 95.9%).

We further investigated the changes of serum CRP levels in childhood epilepsy treated with levetiracetam. As shown in Figure 3, serum CRP level was significantly decreased by the treatment of levetiracetam in childhood epilepsy (pooled SMD: 3.505, 95% CI: 1.638–5.373, z = 3.68, *p* < 0.01). Large between-study heterogeneity was found (τ^2^ = 4.346, Chi^2^ = 97.17, df = 4, *p* < 0.01, I^2^ = 95.9%).

### Publication Bias

**FIGURE 4:**
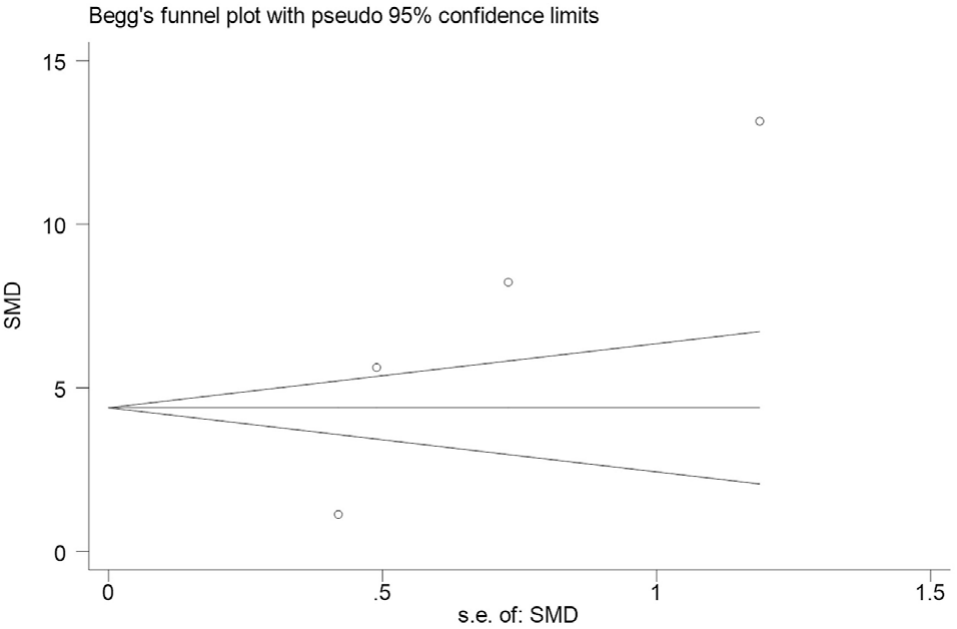
Funnel plot of studies by Stata for four studies included in the meta-analysis. No significant publication bias in the meta-analysis for the difference of serum level of CRP between childhood epilepsy and healthy controls (*p* = 0.108).

**FIGURE 5:**
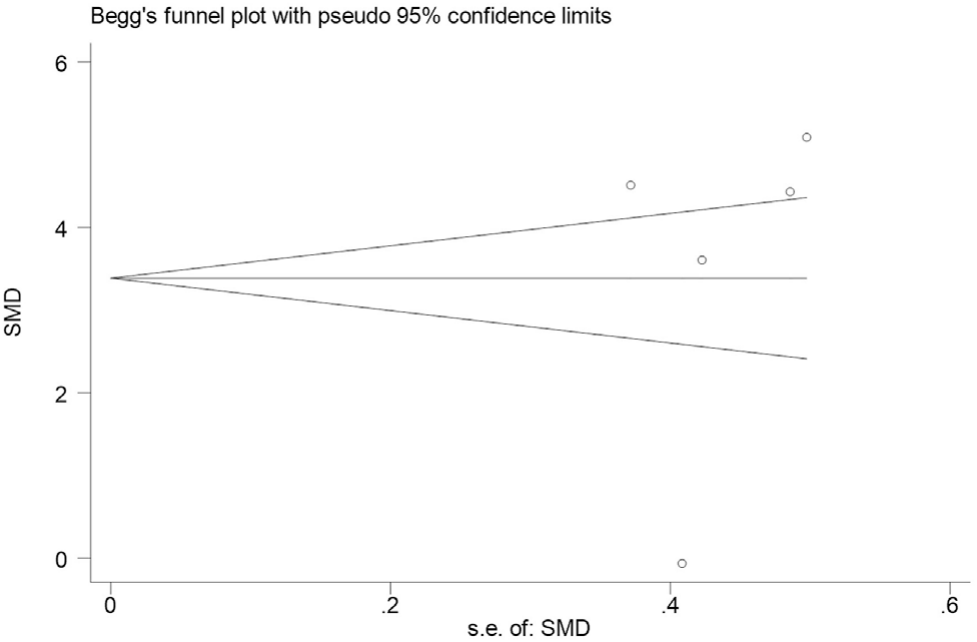
Funnel plot of studies by Stata for five studies included in the meta-analysis. No obvious publication bias was found in the meta-analysis on the changes of serum CRP level in childhood epilepsy treated by levetiracetam (*p* = 0.647).

Subsequently, we conducted an analysis on publication bias for the included articles using funnel plots and Egger’s test for the serum levels of CRP. Although visual inspection of the funnel plot indicated a slightly asymmetrical distribution for studies included in the meta-analysis (Figure 4; Figure 5), the analysis result suggested that there was no significant publication bias in the meta-analysis on the difference of serum level of CRP between children with epilepsy and healthy controls (*p* = 0.108, Figure 4). In addition, no obvious publication bias was found in the meta-analysis on the changes of serum CRP level in children with epilepsy treated by levetiracetam (*p* = 0.647, Figure 5).

## Discussion

Epilepsy is one of the most common neurological diseases, affecting the life of about 50 million people of all ages around the world, however, the incidence of epilepsy in children is the highest (Thijs et al., 2019). In the past decades, therapy of anti-seizure medications has been considered as the main method for treating childhood epilepsy (Johnson and Kaminski, 2020). Since levetiracetam exhibits high safety and efficacy, the application of levetiracetam in childhood epilepsy attracts more and more attentions and has been widely used in clinic practice (Tabrizi et al., 2019; van der Meer et al., 2021). Numerous researches illustrate the role of inflammatory response in the pathogenesis and development of childhood epilepsy (Paudel et al., 2018; Rana and Musto, 2018), however, limited researches report the effect of levetiracetam on serum CRP level in children with epilepsy. In this study, we conducted a meta-analysis to investigate the effect of levetiracetam on serum level of CRP in childhood epilepsy by analyzing eight recent articles. We for the first time demonstrated that levetiracetam decreased serum level of CRP in childhood epilepsy.

Accumulating evidence reveals the role of CRP in a variety of non-inflammatory neurologic diseases, whereas, few studies illustrate the role of CRP in epilepsy. As reported, aging patients with chronic epilepsy showed some abnormal characteristics such as obvious elevation for BMI, hs-CRP, HOMA-IR, glucose and so on (Hermann et al., 2017). Alapirtti et al. found that CRP concentration was significantly higher in patients with refractory focal epilepsy than healthy controls, suggesting the link between inflammation and refractory epilepsy (Alapirtti et al., 2012). A systematic review and meta-analysis also illustrated that, serum CRP level in patients with epilepsy was obviously upregulated compared to the healthy controls (Zhong et al., 2019). In this meta-analysis, we also found that serum CRP levels were notably increased in childhood epilepsy compared with healthy controls (pooled SMD: 6.930, 95% CI: 2.716–11.143, I^2^ = 98.0%, *p* < 0.01). Moreover, no significant publication bias was observed in the meta-analysis (*p* = 0.108). Our meta-analysis further supported current findings that serum CRP was increased in children with epilepsy using a standardized approach with the assessment for the credibility of these findings.

Accumulating evidence shows that chronic inflammatory response is involved in neurodegenerative processes, such as epilepsy. What’s more, inflammation exhibits regulating affect on the pathophysiology of epilepsy of different types (Haghikia et al., 2008). Several *in vitro* and *in vivio* studies demonstrated the effect of levetiracetam on inflammatory response of patients with epilepsy. An *in vitro* study showed that levetiracetam exhibited anti-inflammatory effect on neuroglia via suppressing IL-1β expression in chronic epileptic rats (Kim et al., 2010). Another study on rat revealed that levetiracetam decreased the elevation of IL-1β in M30 co-cultures, showing the anti-inflammatory potentials of levetiracetam as a kind of anti-seizure medications (Prasad et al., 2018). A clinic investigation showed that levetiracetam significantly decreased serum concentrations of hs-CRP, S100B, NPY and GAL, and improved life quality of patients with refractory epilepsy (Chen et al., 2015). The studies included in this meta-analysis also illustrated that levetiracetam significantly decreased serum CRP contents in children with epilepsy (Li, 2012; Jiang, 2013; Nishiyama et al., 2019; Zheng et al., 2019). Therefore, a meta-analysis is necessary to further confirm the effect of levetiracetam on CRP concentration in children with epilepsy. The present study demonstrated that levetiracetam could remarkably decrease serum content of CRP in childhood epilepsy (pooled SMD: 3.505, 95% CI: 1.638–5.373, I^2^ = 95.9%, *p* < 0.01). No publication bias was found in the analysis (*p* = 0.647). Our results further identified a decrease of serum CRP in children with epilepsy treated with levetiracetam.

This study also has some limitations. For instance, the included references in the meta-analysis are limited. Secondly, most studies were collected from China.

In summary, the available evidence indicates that serum contents of CRP are elevated in childhood epilepsy and significantly decreased after the treatment of levetiracetam. These findings might provide convincing evidence for the effect of levetiracetam on serum CRP in childhood epilepsy.

## Data Availability

The original contributions presented in the study are included in the article/Supplementary Material, further inquiries can be directed to the corresponding author.
